# Demographic Disparities in Mpox Vaccination Series Completion, by Route of Vaccine Administration — California, August 9, 2022–March 31, 2023

**DOI:** 10.15585/mmwr.mm7230a4

**Published:** 2023-07-28

**Authors:** Tarek Salih, Josh Vance, Joshua Quint, Brenda Meza, Louise McNitt, Webster U. Lincoln, Robert Schechter

**Affiliations:** ^1^California Department of Public Health; ^2^Immunization Services Division, National Center for Immunization and Respiratory Diseases, CDC.

**Summary**

**What is already known about this topic?**

Demographic disparities among persons completing the 2-dose mpox vaccination series have been previously described.

**What is added by this report?**

California residents who received their first dose of mpox vaccine by intradermal or subcutaneous administration had comparable 2-dose series completion rates (60.2% and 58.8%, respectively). Similar series completion rates by route of administration were observed across all race and ethnicity groups, persons aged 18–64 years, community health conditions, and persons assigned male sex at birth.

**What are the implications for public health practice?**

Route of administration of the first dose was not associated with lower overall 2-dose series completion rates. Continued efforts are needed to ensure persons at risk for mpox receive both recommended doses.

## Abstract

In August 2022, the Food and Drug Administration authorized JYNNEOS vaccine (modified vaccinia Ankara vaccine, Bavarian Nordic), a 2-dose series used for the prevention of *Monkeypox virus* infection, to be administered via a dose-sparing intradermal route, in addition to the previously authorized subcutaneous route. The California Department of Public Health investigated whether demographic disparities in vaccination series completion varied by route of administration of the recipient’s first dose. Among California residents who received their first dose during August 9, 2022–March 31, 2023, a total of 59.8% received a second dose. Series completion was highest among non-Hispanic White persons (64.1%), persons aged ≥65 years (72.6%), and adults with male sex assignment at birth (62.1%); series completion was lowest among non-Hispanic Black or African American persons (51.3%), persons aged 18–24 years (42.9%), and adults assigned female sex at birth (42.8%). When the first dose was received by subcutaneous administration, overall series completion was 58.8% compared with 60.2% when the first dose was administered intradermally. Odds of series completion across all race and ethnicity groups, persons aged 18–64 years, community health conditions, and persons assigned male sex at birth were not greater when the first dose was administered subcutaneously compared with intradermally. Intradermal use of JYNNEOS vaccine did not lower overall 2-dose series completion rates. Continued efforts are needed to ensure persons at risk for *Monkeypox virus* infection receive both recommended doses.

## Introduction

In response to the 2022 U.S. mpox outbreak, CDC and the Administration for Strategic Preparedness and Response initiated distribution of JYNNEOS smallpox and mpox vaccine, licensed in the United States as a 2-dose series, with doses administered 28 days apart (*1*). During May 26, 2022–August 8, 2022, the vaccine was exclusively administered via subcutaneous (SC) injection of a 0.5 mL dose (*2*). On August 9, 2022, the Food and Drug Administration authorized a dose-sparing 0.1 mL intradermal (ID) injection (*3*). Despite increased availability resulting from ID administration and efforts to improve access while the outbreak evolved, 2-dose vaccination series completion among California residents was 64.5% overall^†^ and varied across demographic groups (*2*). Concerns were raised that ID administration of the first dose might lead to lower series completion among persons at risk for scarring or keloid formation (*4*).

## Methods

Persons aged ≥18 years with documentation of receipt of ≥1 dose of JYNNEOS vaccine reported to the California Immunization Registry during August 9, 2022–March 31, 2023, were included. The starting date of August 9, 2022, was used to restrict the analysis to the period after authorization of ID administration of JYNNEOS. Descriptive statistics were calculated for persons who had received ≥1 reported dose of JYNNEOS for which the route of administration of the first dose was recorded, and results were stratified by demographic groups. Persons who had received 2 doses of JYNNEOS vaccine were included if a minimum of 24 days^§^ separated the first and second dose and the second dose was reported on or before April 30, 2023. Odds ratios (ORs) and 99% Wald CIs were estimated using logistic regression to assess differences in series completion overall and by route of administration of the first dose^¶^ stratified by race and ethnicity, age group, community health conditions (using Healthy Places Index [HPI] quartiles ranked from 1 [least healthy] to 4 [healthiest])** (*5*), and sex assignment at birth.^††^ Similarly, to ascertain whether series completion was affected by policy changes, vaccine supply, and mpox incidence over time, completion rates by month of receipt of the first dose were assessed. SAS statistical software (version 9.4; SAS Institute) was used for all analyses. This activity was reviewed by CDC and was conducted consistent with applicable federal law and CDC policy.^§§^

## Results

Among 119,345 California residents who received their first JYNNEOS dose during August 9, 2022–March 31, 2023, a total of 71,317 (59.8%) completed the 2-dose series (Table). Persons who were assigned female sex at birth (42.8%) had lower odds of returning for a second dose than did those assigned male sex at birth (62.1%). Compared with the odds of completing the series among non-Hispanic White (White) persons (64.1%), the odds were lower among those who were non-Hispanic Black or African American (Black) (51.3%), Hispanic or Latino (Hispanic) (56.6%), non-Hispanic Asian (Asian) (60.8%), and non-Hispanic multiracial or other race (58.1%). Similarly, the odds of completing the 2-dose series were lower among those aged 45–54 years (64.5%), 35–44 years (58.9%), 25–34 years (51.7%), and 18–24 years (42.9%), compared with those among aged ≥65 years (72.6%), but were similar among those aged 55–64 years (70.0%). The odds of receiving a second dose, when compared to persons in HPI quartile 4 (58.8%), were similar among persons living in quartile 1 (56.9%), but higher among persons living in quartile 2 (62.9%) and 3 (62.4%).

**TABLE:** Percentage and odds ratios for completing the mpox vaccine series, by route of administration of first dose and demographic subgroup — California, August 9, 2022–March 31, 2023

Characteristic	First doses, route of administration, no.			Completed series, route of first dose administration, no. (%)*			Odds ratio (99% CI)^¶^,**
	Total^†^	SC	ID	Total (SC or ID)^§^	SC	ID	
**Total**	**119,345**	**35,862**	**83,483**	**71,317 (59.8)**	**21,084 (58.8)**	**50,233 (60.2)**	**NA**
**Sex assigned at birth**							
Female	**13,446**	4,086	9,360	**5,759 (42.8)**	1,503 (36.8)	4,256 (45.5)	0.69 (0.66–0.72)
Male	**105,366**	31,614	73,752	**65,387 (62.1)**	19,521 (61.7)	45,866 (62.2)	Ref
Unknown or other^††^	**533**	162	371	**171 (32.0)**	60 (37.0)	111 (29.9)	NA^§§^
**Race and ethnicity**							
Asian, NH	**14,284**	4,729	9,555	**8,686 (60.8)**	2,845 (60.2)	5,841 (61.1)	0.95 (0.91–0.99)
Black or African American, NH	**8,828**	3,010	5,818	**4,531 (51.3)**	1,536 (51.0)	2,995 (51.5)	0.80 (0.76–0.84)
White, NH	**53,059**	15,237	37,822	**33,992 (64.1)**	9,655 (63.4)	24,337 (64.3)	Ref
Hispanic or Latino	**31,270**	9,204	22,066	**17,691 (56.6)**	5,081 (55.2)	12,610 (57.1)	0.88 (0.86–0.91)
Multiracial/Other	**9,698**	3,003	6,695	**5,632 (58.1)**	1,742 (58.0)	3,890 (58.1)	0.91 (0.87–0.95)
Unknown	**2,206**	679	1,527	**785 (35.6)**	225 (33.1)	560 (36.7)	NA^§§^
**Age group, yrs**							
18–24	**8,950**	2,607	6,343	**3,838 (42.9)**	1,069 (41.0)	2,769 (43.7)	0.59 (0.55–0.63)
25–34	**32,963**	10,588	22,375	**17,032 (51.7)**	5,427 (51.3)	11,605 (51.9)	0.71 (0.68–0.74)
35–44	**26,453**	8,384	18,069	**15,568 (58.9)**	5,013 (59.8)	10,555 (58.4)	0.81 (0.77–0.85)
45–54	**19,767**	6,017	13,750	**12,742 (64.5)**	3,866 (64.3)	8,876 (64.6)	0.89 (0.85–0.93)
55–64	**20,390**	5,661	14,729	**14,280 (70.0)**	3,912 (69.1)	10,368 (70.4)	0.96 (0.92–1.01)
≥65	**10,822**	2,605	8,217	**7,857 (72.6)**	1,797 (69.0)	6,060 (73.7)	Ref
**Healthy Places Index quartile** ^§§^							
1 (least healthy)	**21,505**	5,697	15,808	**12,246 (56.9)**	3,160 (55.5)	9,086 (57.5)	0.97 (0.93–1.00)
2	**25,127**	6,655	18,472	**15,809 (62.9)**	4,056 (60.9)	11,753 (63.6)	1.07 (1.04–1.10)
3	**29,234**	8,404	20,830	**18,254 (62.4)**	5,181 (61.6)	13,073 (62.8)	1.06 (1.03–1.09)
4 (healthiest)	**40,548**	14,399	26,149	**23,840 (58.8)**	8,394 (58.3)	15,446 (59.1)	Ref
Unknown	**2,931**	707	2,224	**1,168 (39.8)**	293 (41.4)	875 (39.3)	NA^§§^

**Abbreviations:** ID = intradermal; NA = not applicable; NH = non-Hispanic; Ref = referent group; SC = subcutaneous.

Persons who received a second dose by same route of administration as that of the first dose.

^†^ Route of administration was not reported for every first dose.

^§^ Irrespective of route of administration of first dose.

^¶^ Within the same column (e.g., comparing persons aged >64 years with other age groups).

** Odds of returning for a second dose, irrespective of route of administration.

^††^ Data submitted to California Immunization Registry do not include sexual orientation or gender identity information and are generally based on the sex or gender entered at the time of the person’s first dose of any vaccine; however, some records have been updated to reflect a person’s gender identity as being “other” or “nonbinary.”

^§§^ There are many reasons why demographic variables might be unknown and statistical findings could be biased. Thus, odds ratios were not calculated when demographic variables were unknown.

^¶¶^ Healthy Places Index quartiles are based on data from 25 identified key drivers of health and life expectancy at birth. Each county and zip code are assigned to a quartile based on these 25 measures, ranked from 1 (least healthy) to 4 (healthiest). https://map.healthyplacesindex.org

**FIGURE 1:**
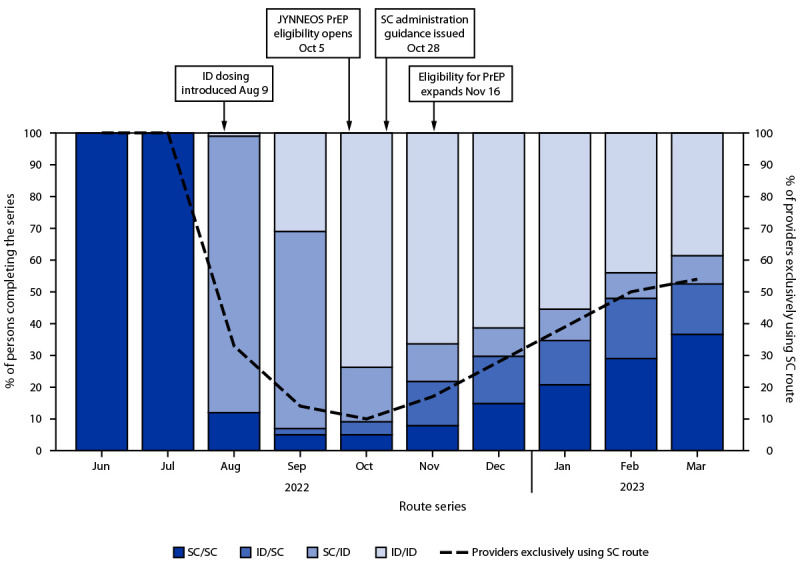
Route of JYNNEOS vaccine administration used by persons completing the 2-dose mpox vaccine series, by month of administration of the second dose and proportion of health care providers exclusively offering subcutaneous administration*^,†,§,¶^ — California, June 2022–March 2023 **Abbreviations: **CDPH = California Department of Public Health; FDA = Food and Drug Administration; ID = intradermal; PrEP = preexposure prophylaxis; SC = subcutaneous. * After FDA’s authorization of ID administration on August 9, 2022, CDPH immediately recommended that persons receive mpox vaccine via ID route, with exceptions for persons with history of keloid formation and persons aged <18 years. Previously, only SC administration of JYNNEOS vaccine was authorized. ^†^ On October 5, 2022, CDPH released guidance to local health jurisdictions and medical providers offering JYNNEOS vaccine to expand the pool of persons eligible to those at risk of infection (i.e., preexposure prophylaxis). Before this date, CDPH guidance recommended JYNNEOS be used to vaccinate persons exposed to *Monkeypox virus* (i.e., postexposure prophylaxis) or those with the greatest risk of infection, including persons who frequented venues where *Monkeypox virus* had been circulating (i.e., expanded postexposure prophylaxis). ^§^ As incidence of mpox decreased and supply became ample, CDPH issued guidance on October 28, 2022, permitting provider and patient discretion regarding route of administration, allowing for vaccine to be administered either intradermally or subcutaneously. ^¶^ On November 16, 2022, CDPH again issued guidance to expand vaccine eligibility to all persons who might be at risk for *Monkeypox virus* exposure and persons who request vaccination. ** The proportion of providers across California who were exclusively administering vaccine to patients via SC route began to steadily increase in November 2022. This change coincides with the proportion of persons who were completing the series (i.e., second dose) with a subcutaneously administered vaccine dose. The surveillance data used in this analysis cannot differentiate between persons who requested vaccine to be administered subcutaneously versus those who were seen by providers who offered vaccine exclusively to patients via SC route.

**FIGURE 2:**
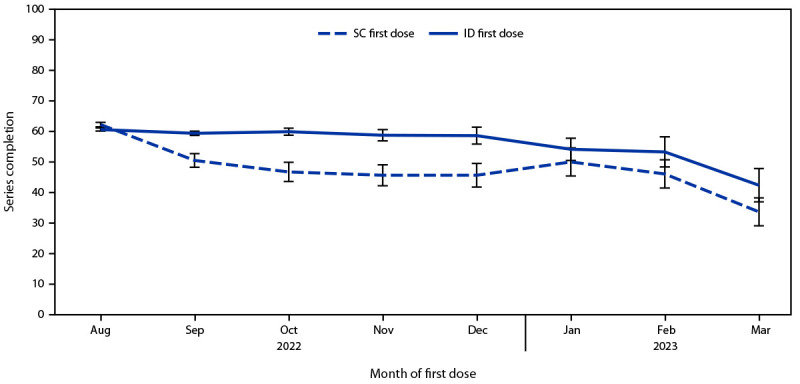
Percentage of persons completing the 2-dose mpox vaccination series, by administration route of the first dose and month of administration of the first dose — California, August 9, 2022–March 31, 2023*^,† ^ **Abbreviations: **ID = intradermal; SC = subcutaneous. * The percentage of all persons in any given month who completed the vaccine series ≥24 days after their first dose and received their second dose no later than April 30, 2023, stratified by the route of administration of the first dose. For example, 51% and 59% of persons whose first dose was administered by SC and ID route, respectively, in September 2022 completed the vaccine series no later than April 30, 2023. ^†^ August includes August 9–31, 2022, when both ID and SC administration were authorized. August 1–8, 2022, was excluded.

Overall, among 119,345 first doses administered since August 9, 2022, 83,483 (70.0%) were administered by the ID route and 35,862 (30.0%) by the SC route.^¶¶^ The proportion of ID doses began to decline in October 2022 (Figure 1). Despite this decline and concerns regarding ID administration, the proportion of persons receiving a second dose after ID administration of the first dose (60.2%) was not lower than the proportion of those who received second dose after SC administration of the first dose (58.8%) (Supplementary Table, https://stacks.cdc.gov/view/cdc/131259). This finding was consistent among Asian, Black, White, Hispanic, and multiracial or other race persons; persons aged 18–64 years; persons in all four HPI quartiles (*5*); and persons assigned male sex at birth. Among persons aged ≥65 years and those assigned female sex assignment at birth, completion of the series was less likely after SC administration of the first dose. Disaggregation of the data by month found that completion rates among persons receiving their first dose during August 9, 2022–August 31, 2022, were 66.3% and 62.0% among those who received the vaccine by SC and ID administration, respectively, compared with 47.1% and 58.9%, respectively, among those who completed the vaccination series during September 1, 2022–March 31, 2023 (Figure 2) (Supplementary Figure, https://stacks.cdc.gov/view/cdc/131001).***

## Discussion

Despite concern that reactogenicity related to ID administration of JYNNEOS vaccine might lead to lower series completion rates, analysis of California Immunization Registry data found comparable series completion rates irrespective of the route of administration of the first dose. Lower overall series completion, irrespective of route of administration, was observed among persons assigned female sex at birth, certain racial and ethnic groups, and younger persons. In no demographic group was series completion more likely when the first dose was administered by the SC route compared with ID although persons with female sex assignment at birth and those aged ≥65 years were more likely to complete the series when the first dose was administered via the ID route.

Noninferiority in immunogenicity between ID and standard administration routes for influenza, rabies, and hepatitis B vaccinations has been demonstrated (*6*). JYNNEOS vaccine effectiveness against medically attended mpox has been reported to be as high as 86% for 2 doses and 75% for 1 dose (*7*). When comparing route of administration, no significant differences in vaccine effectiveness have been demonstrated to date (*7*,*8*). Although ID vaccine recipients have reported differences in period of swelling after vaccination (*9*), a CDC analysis of data from the Vaccine Adverse Event Reporting System and the Vaccine Safety Datalink found no significant differences in the prevalence of adverse events reported for ID versus SC administration of JYNNEOS (*10*).

### Limitations

The findings in this report are subject to at least five limitations. First, the California Immunization Registry does not include data on behavioral risk; thus, certain risk factors that might have affected series completion could not be evaluated. Second, ID administration was introduced after many persons (i.e., those vaccinated during May 26–August 8, 2022) had already received their first dose. Among these early vaccine recipients, 75.1% completed the series; whether their series completion rates would have been similar had their first dose been administered intradermally is not known. Third, declining case rates, starting in August 2022, might have led to reduced interest in mpox vaccination and could have affected self-perceived risk and the need for a second dose in certain populations. Fourth, these data only determine odds of completing the vaccination series and do not consider persons who chose not to initiate the series. Finally, California-specific data might not be generalizable to other jurisdictions.

### Implications for Public Health Practice

JYNNEOS vaccination series completion in California was not affected by route of first dose administration. Issues including access to vaccination, assessment of patient risk, and communication to disaffected populations by trusted messengers might be considered for future studies on disparities in vaccine acceptance. It remains important that health care providers discuss the benefits and risks associated with different administration routes with patients and ensure that patients understand the importance of completing the 2-dose JYNNEOS vaccination series. Similarly, contacting patients overdue for their second dose, particularly in groups with the lowest odds of series completion (e.g., persons aged 18–24 years), might help improve vaccination rates. Focused outreach, culturally sensitive messaging, and direct engagement by trusted messengers to groups disproportionately represented in mpox cases (e.g., Black and Hispanic persons) remain essential to ensuring that patients receive the benefit of a complete 2-dose series, and ultimately, to preventing future outbreaks.

## Footnotes

* These authors contributed equally to this report.

† Among California residents who received a first dose of JYNNEOS during May 26, 2022–March 31, 2023, 64.5% completed the vaccination series by April 30, 2023. During the study period (August 9, 2022–March 31, 2023), 59.8% completed the vaccination series by April 30, 2023.

§ JYNNEOS is licensed as a 2-dose vaccine, with doses recommended to be given 28 days apart; however, a minimum interval of 24 days between doses is acceptable. https://www.cdc.gov/poxvirus/mpox/interim-considerations/jynneos-vaccine.html#dosing

¶ Data collected in the California Immunization Registry do not differentiate between patients returning for their second dose who 1) might have requested one route of administration versus the other, 2) were recommended by their provider to receive one route versus the other, or 3) visited a provider exclusively offering one route type. Because of these limitations, this analysis did not consider route of administration of the second dose when assessing whether administration route of the first dose acted as a deterrent to vaccine series completion.

** HPI quartiles are based on data from 25 identified key drivers of health and life expectancy at birth. Each county and zip code are assigned to a quartile based on these 25 measures, ranked from 1 (least healthy) to 4 (healthiest). https://map.healthyplacesindex.org

†† Data submitted to the California Immunization Registry do not currently include sexual orientation or gender identity information and are generally based on the sex or gender entered at the time of the person’s first dose of any vaccine; however, some records have been updated to reflect a person’s gender identity as being “other” or “nonbinary.”

§§ 45 C.F.R. part 46.102(l)(2), 21 C.F.R. part 56; 42 U.S.C. Sect. 241(d); 5 U.S.C. Sect. 552a; 44 U.S.C. Sect. 3501 et seq.

¶¶ Before Food and Drug Administration authorization of ID administration of JYNNEOS vaccine on August 9, 2022, an additional 53,892 total doses were administered by the SC route.

*** A decreasing trend in second dose return rate among persons receiving their first dose subcutaneously during June–September 2022 has been observed and might coincide with declining mpox incidence and changing demographic characteristics of vaccine recipients.
